# Glycomic analyses of ovarian follicles during development and atresia

**DOI:** 10.1016/j.matbio.2011.10.002

**Published:** 2012-01

**Authors:** Nicholas Hatzirodos, Julie Nigro, Helen F. Irving-Rodgers, Aditya V. Vashi, Katja Hummitzsch, Bruce Caterson, Thomas R. Sullivan, Raymond J. Rodgers

**Affiliations:** a Research Centre for Reproductive Health, Discipline of Obstetrics and Gynaecology, School of Paediatrics and Reproductive Health, Robinson Institute, University of Adelaide, SA, 5005, Australia; b CSIRO, Materials Science and Engineering, Clayton, Victoria 3168, Australia; c Department of Anatomy and Developmental Biology, Monash University, Clayton, Victoria 3168, Australia; d Cancer Program, Institute of Health and Biomedical Innovation, Queensland University of Technology, Kelvin Grove, QLD, 4059, Australia; e Connective Tissue Biology Laboratories, Cardiff School of Biosciences, Cardiff University, Cardiff, UK; f Data Management and Analysis Centre, Discipline of Public Health, University of Adelaide, SA, 5005, Australia

**Keywords:** chondroitin sulfate, granulosa cells, hyaluronan, heparan sulfate, inter-α-trypsin inhibitor, thecal cells, versican

## Abstract

To examine the detailed composition of glycosaminoglycans during bovine ovarian follicular development and atresia, the specialized stromal theca layers were separated from the stratified epithelial granulosa cells of healthy (n = 6) and atretic (n = 6) follicles in each of three size ranges: small (3–5 mm), medium (6-9 mm) and large (10 mm or more) (n = 29 animals). Fluorophore-assisted carbohydrate electrophoresis analyses (on a per cell basis) and immunohistochemistry (n = 14) were undertaken. We identified the major disaccharides in thecal layers and the membrana granulosa as chondroitin sulfate-derived ∆uronic acid with 4-sulfated *N*-acetylgalactosamine and ∆uronic acid with 6-sulfated *N*-acetylgalactosamine and the heparan sulfate-derived Δuronic acid with *N*-acetlyglucosamine, with elevated levels in the thecal layers. Increasing follicle size and atresia was associated with increased levels of some disaccharides. We concluded that versican contains 4-sulfated *N*-acetylgalactosamine and it is the predominant 4-sulfated *N*-acetylgalactosamine proteoglycan in antral follicles. At least one other non- or 6-sulfated *N*-acetylgalactosamine proteoglycan(s), which is not decorin or an inter-α-trypsin inhibitor family member, is present in bovine antral follicles and associated with hitherto unknown groups of cells around some larger blood vessels. These areas stained positively for chondroitin/dermatan sulfate epitopes [antibodies 7D4, 3C5, and 4C3], similar to stem cell niches observed in other tissues. The sulfation pattern of heparan sulfate glycosaminoglycans appears uniform across follicles of different sizes and in healthy and atretic follicles. The heparan sulfate products detected in the follicles are likely to be associated with perlecan, collagen XVIII or betaglycan.

## Highlights

► In ovarian follicles the major chondroitin sulfates are ∆uronic acid with 4- or 6-sulfated *N*-acetylgalactosamine. ► Versican is the predominant 4-sulfated *N*-acetylgalactosamine proteoglycan in follicles. ► An unidentified non- or 6-sulfated *N*-acetylgalactosamine proteoglycan is also present in follicles. ► 6-sulfated *N*-acetylgalactosamine epitopes [antibodies 7D4, 3C5, 4C3 and 3B3(+)] identify a potential stem cell niche. ► The major heparan sulfate are Δuronic acid with *N*-acetlyglucosamine, and the sulfation is uniform in the follicles examined.

## 1 Introduction

The mammalian adult ovary contains a reserve of inactive primordial follicles which develop during fetal life. In many species this development is completed before birth. Each inactive primordial follicle contains a small non-growing oocyte and a layer of non-dividing pre-granulosa cells encapsulated by the follicular basal lamina. Once the reserve of primordial follicles is established a number of these primordial follicles become activated on a continuing basis until the reserve is depleted at menopause. On activation the oocyte commences growing while the granulosa cells begin to divide. As the granulosa cells divide, the number of layers of cells (called the membrana granulosa or follicular epithelium) around the oocyte increases, and the follicular basal lamina expands. Later in development a fluid-filled cavity or antrum forms in the middle of the follicle and specialized stromal layers, the theca interna and externa, develop outside the follicular basal lamina. Theca cells and granulosa cells are initially regulated separately by the hormonal gonadotropins and they cooperatively produce the steroid hormone estradiol. Their ability to do so increases as the follicles enlarge, and only follicles that reach the stage of having a large antrum, and in the follicular wave following regression of corpora lutea, can ovulate an oocyte in response to the surge release of luteinizing hormone. Once activated, follicles that do not ovulate undergo atresia and regression instead. This is the fate of the vast majority of follicles and serves to reduce the number of oocytes ovulated and to control the timing of ovulation within a reproductive cycle. Atresia of antral follicles initially involves death of granulosa cells and subsequently thecal cells and oocytes and it involves apoptosis, autophagy and later necrosis and resorption of cell debris by macrophages. Development and atresia of mammalian ovarian follicles and oocytes is therefore a complex process involving extensive tissue growth and remodelling, fluid accumulation, and replication, specialization, differentiation and death of cells.

Proteoglycans (PGs) are ubiquitous molecules of extracellular matrices that have been implicated in developmental processes in a variety of other tissues (Hardingham and Fosang, 1992). The PGs consist of glycosaminoglycans covalently attached to a protein core. The glycosaminoglycans consist of chains containing repeating disaccharide units that vary in composition and sulfation pattern, depending on their deposition and function (Wight, 2002; Habuchi et al., 2004). Some glycosaminoglycans have been identified in the ovarian follicular fluid of pigs (Ax and Ryan, 1979; Yanagishita et al., 1979; Sato et al., 1990), cows (Lenz et al., 1982; Bellin et al., 1983; Grimek et al., 1984; Bellin and Ax, 1987a,b), humans (Bellin et al., 1986; Eriksen et al., 1994, 1997, 1999) and rats (Gebauer et al., 1978; Mueller et al., 1978). The predominant glycosaminoglycans in bovine and porcine follicular fluid are dermatan sulfate (DS) and chondroitin sulfate (CS) (Yanagishita et al., 1979; Bellin et al., 1983). The CS/DS-containing glycosaminoglycans were shown to be attached to a protein in bovine follicles while the heparan sulfate (HS) glycosaminoglycans were not bound to protein (Grimek and Ax, 1982). The concentration of glycosaminoglycans in bovine follicular fluid varied with size and health of the developing follicles (Grimek and Ax, 1982; Bellin and Ax, 1987b). The concentration of CS is higher in the follicular fluid of small-antral follicles as compared with large antral follicles (Grimek and Ax, 1982). The concentration of CS was also reported to vary with the health of the ovarian follicle (Bellin and Ax, 1984).

A number of PGs have been identified in follicles. Versican is a large CS PG identified in extracts of bovine follicles (McArthur et al., 2000), follicular fluid of non-ovulating (Clarke et al., 2006) and ovulating follicles (Eriksen et al., 1999), in the thecal layer adjacent to the follicular basal lamina (McArthur et al., 2000; Irving-Rodgers and Rodgers, 2006) and in the follicular membrana granulosa (McArthur et al., 2000; Irving-Rodgers et al., 2004). Versican is also expressed by rat and mouse granulosa cells (Russell et al., 2003). Decorin is a small leucine-rich repeat PG with a CS/DS glycosaminoglycan and was identified in extracts of bovine follicles (McArthur et al., 2000) and localized strongly to collagen-rich bovine ovarian tunica albuginea and less strongly throughout the ovarian stroma (Irving-Rodgers and Rodgers, 2007). Decorin is also located in the thecal layers of antral follicles, with increased amounts in the theca externa compared with the theca interna (Irving-Rodgers and Rodgers, 2007). Bikunin is a CS PG present in bovine and porcine follicular fluids as a component of inter-α-trypsin inhibitor, pre-α-trypsin inhibitor, and inter-α-like trypsin inhibitor (Nagyova et al., 2004; Clarke et al., 2006) and is likely to be derived from the serum of the thecal vasculature (Rodgers and Irving-Rodgers, 2010a). Immunoreactivity using antibody against inter-α-like trypsin inhibitor localizes to cumulus cells, antrally-situated granulosa cells and to the stromal side of the follicular basal lamina in mouse follicles (Irving-Rodgers and Rodgers, 2005). Hyaluronan (HA) also localizes to these same regions in bovine follicles (Irving-Rodgers and Rodgers, 2005).

A number of HS PGs have been identified in the ovary. Perlecan is present in the follicular basal laminas and sub-endothelial basal laminas in the thecal layers of bovine follicles at the antral stages in both healthy and atretic follicles (McArthur et al., 2000). In mice, however, perlecan is present in the follicular basal lamina at all stages of follicular development and in the thecal sub-endothelial basal laminas (Irving-Rodgers et al., 2010). Collagen type XVIII is also present in the follicular basal laminas and sub-endothelial basal laminas in bovine antral follicles (Irving-Rodgers and Rodgers, 2006). In mice, collagen type XVIII is present in primordial and primary follicles but is limited to some preantral and antral follicles and is not in the thecal sub-endothelial basal laminas (Irving-Rodgers et al., 2010). Glypican-6 mRNA has been identified but not localized in human ovaries (Veugelers et al., 1999). Betaglycan in bovine antral follicles is significantly higher in the theca than in granulosa cells and positively correlated with increasing follicles size, at least in the thecal layer (Glister et al., 2010).

Ovarian follicles have an additional specialized basal lamina-type of matrix, called focal intra-epithelial matrix (focimatrix) that is composed of aggregates of basal lamina deposited between the stratified granulosa cells in the follicle wall (Irving-Rodgers et al., 2004, 2006). It appears late in follicular development, increases in abundance as follicles enlarge to pre-ovulatory sizes (Irving-Rodgers et al., 2004, 2006) and is degraded at ovulation (Irving-Rodgers and Rodgers, 2006). Focimatrix exists in cattle (Irving-Rodgers et al., 2004), sheep (Huet et al., 1997), humans (Yamada et al., 1999; Alexopoulos et al., 2000) and mice (Nakano et al., 2007; Irving-Rodgers et al., 2010). It has been suggested to be involved in an epithelial-mesenchymal transition of granulosa cells into luteal cells (Irving-Rodgers et al., 2004) and recent evidence suggests that it may be important for regulating the expression of key enzymes needed for synthesis of progesterone and estradiol (Irving-Rodgers et al., 2009; Matti et al., 2010). Bovine and murine focimatrix contains, among other basal lamina components, perlecan and collagen XVIII (Irving-Rodgers et al., 2004; Irving-Rodgers and Rodgers, 2006; Irving-Rodgers et al., 2010).

These previous studies demonstrate that PGs are present and dynamically regulated during follicular development and in atresia. They do not, however, provide quantitative data on the composition of glycosaminoglycans necessary to fully determine their role(s) in follicle development. We therefore isolated the stromal thecal layers and epithelial granulosa cells of antral follicles at three different sizes, and hence of different developmental stages, and also from atretic follicles to determine delta-disaccharide composition of the CS, HS and HA using fluorophore-assisted carbohydrate electrophoresis. In addition we immunolocalized a number of different epitopes of CS (Sorrell et al., 1990) and two CS PGs, versican and inter-α-trypsin inhibitor, in ovaries. To best illustrate the location of these epitopes the staining was combined with immunostaining of known markers of different cell types within the ovary.

## 2 Results

### 2.1 Fluorophore-assisted carbohydrate electrophoresis analyses

**Fig. 1:**
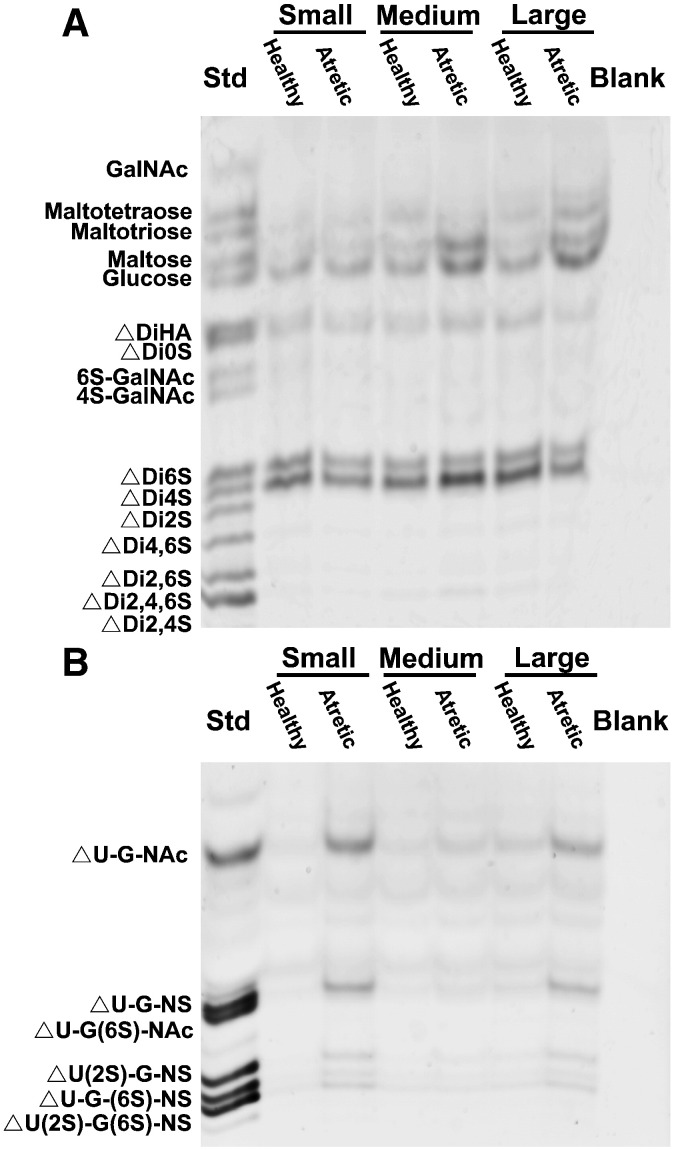
Examples of fluorophore-assisted carbohydrate electrophoresis identifying saccharides following enzymatic digestion of theca interna isolated from both healthy and atretic follicles of different sizes (small, medium and large) with either (A) hyaluronidase SD and chondroitinase ABC or (B) heparinase and heparitinase I and II. In (A) glucose, maltose, maltotriose and maltotetraose which are not the products of hyaluronidase SD and chondroitinase ABC digestions are also visible. In each gel sacharide standards (Std) and a control without sample but still reacted with 2-aminoacridone (Blank) were included. The abbreviated names of each saccharide are listed in full in Table 1. An equivalent amount of each sample containing 3 μg of DNA was loaded per lane.

Table 1 lists the abbreviations used to describe each dissacharide and Fig. 1 shows examples of fluorophore-assisted carbohydrate electrophoresis identifying saccharides following enzymatic digestion of theca isolated from both healthy and atretic follicles of different sizes (small, medium and large) with either hyaluronidase SD and chondroitinase ABC or heparinase and heparitinase I and II. Glucose, maltose, maltotriose and maltotetraose which are not the products of hyaluronidase SD and chondroitinase ABC digestions were also present in the samples (Fig. 1A). They were not quantitated as they may not have been quantitatively precipitated in the procedure. Comparisons were made on the basis of equivalent amounts of DNA which equates to a per cell basis, and not on a per volume of tissue basis. For some molecules, results were not normally distributed due to multiple values falling below the limit of detection. Since these values were not able to be transformed to achieve a normal distribution, non-parametric analyses were conducted. In these cases when comparing two groups a Wilcoxon's signed rank test, the non-parametric equivalent of a paired Student's *t* test, was used and when comparing more than two groups a Kruskal Wallis test, the non-parametric equivalent of a one-way ANOVA, was carried out. Similarly for correlation analyses the non-parametric Spearman's correlation coefficients were calculated.

Of the disaccharides derived from CS the order of abundance in thecal tissue was ∆Di4S, ∆Di6S and ∆Di0S; with very low levels of ∆Di4,6S and ∆Di2,4,6S and in granulosa cells ∆Di4S, ∆Di6S were present in similar amounts with very low levels of ∆Di0S ∆Di4,6S and ∆Di2,4,6S in granulosa cells (Table 1). The levels of ∆Di0S, ∆Di4S, ∆Di6S were all significantly elevated in the thecal tissue compared to granulosa cells (Table 1). In granulosa cells ∆Di4S was significantly elevated in medium and large follicles (Table 2) and atretic follicles (Table 3). Atretic follicles also had significantly elevated levels of ∆Di4,6S in granulosa cells (Table 3). The levels of ∆Di4S and ∆Di4,6S were correlated in granulosa cells (Supplemental Table 1). In thecal cells the levels of all five ∆Di0S, ∆Di4S, ∆Di6S, ∆Di4,6S, ∆Di2,4,6S were relatively unchanged in healthy and atretic follicles (Table 5) and in follicles of different sizes (Table 4), except for ∆Di6S which was significantly lower in medium-sized follicles compared with small and large follicles. In thecal cells the levels of ∆Di4S correlated with both ∆Di6S and ∆Di4,6S and the levels of ∆Di4,6S significantly correlated with ∆Di2,4,6S in thecal tissue (Supplemental Table 2) and in the combined data from both granulosa cells and thecal tissue (Supplemental Table 3).

There was no significant change in ∆DiHA associated with healthy and atretic follicles or between follicles of different sizes in either granulosa cells (Tables 2 and 3) or thecal tissue (Tables 4 and 5) nor between granulosa cells and thecal tissue (Table 1). In granulosa cells the levels of ∆DiHA correlated with ∆Di4S (Supplemental Table 1) and in thecal tissue with each of ∆Di4S, ∆Di6S and ∆Di4,6S (Supplemental Table 2) and with ∆Di2,4,6S in the combined data from both thecal and granulosa cells (Supplemental Table 3).

Of the saccharides derived from HS, ΔU-G-NAc, ΔU-G-NS, ΔU(2S)-G-NS and ΔU(2S)-G(6S)-NS were significantly elevated in thecal tissue compared to granulosa cells; the predominant ones in order were ΔU-G-NAc, ΔU-G-NS and ΔU(2S)-G(6S)-NS (Table 1). In granulosa cells the levels of ΔU-G-NAc and ΔU-G-NS were significantly elevated in medium and large-sized follicles (Table 2) and in atretic follicles (Table 3); as were ΔU(2S)-G-NS and ΔU(2S)-G(6S)-NS (Table 3). Nearly all the saccharides derived from HS were significantly correlated with each other in granulosa cells, except ΔU-G-NS and ΔU(2S)-G-NS, and ΔU-G(6S)-NAc and ΔU(2S)-G(6S)-NS (Supplemental Table 1). In thecal tissue the levels of ΔU-G(6S)-NAc, ΔU-G-(6S)-NS, ΔU(2S)-G-NS and ΔU(2S)-G(6S)-NS were significantly elevated in large follicles (Table 4) and the levels of ΔU-G-NAc, ΔU-G-NS, ΔU-G-(6S)-NS, and ΔU(2S)-G-NS were significantly elevated in atretic follicles (Table 5). Nearly all the saccharides derived from HS were significantly correlated with each other in thecal tissue except for ΔU-G-NS and ΔU-G(6S)-NAc and ΔU-G(6S)-NAc and ΔU-G-(6S)-NS (Supplemental Table 2). Combining data from granulosa cells and thecal tissue, nearly all the saccharides derived from HS were significantly correlated with each other except ΔU-G(6S)-NAc and ΔU(2S)-G-NS (Supplemental Table 3). There was a number of other correlations in addition to those reported above (Supplemental Tables 1, 2 and 3), however, of potential significance are the correlations between ∆Di4S and ΔU-G-NAc, ΔU-G-NS, ΔU-G-(6S)-NS, ΔU(2S)-G-NS and ΔU(2S)-G(6S)-NS in granulosa cells (Supplemental Table 1).

### 2.2 Immunohistochemical localization of CS epitopes and versican

**Fig. 2:**
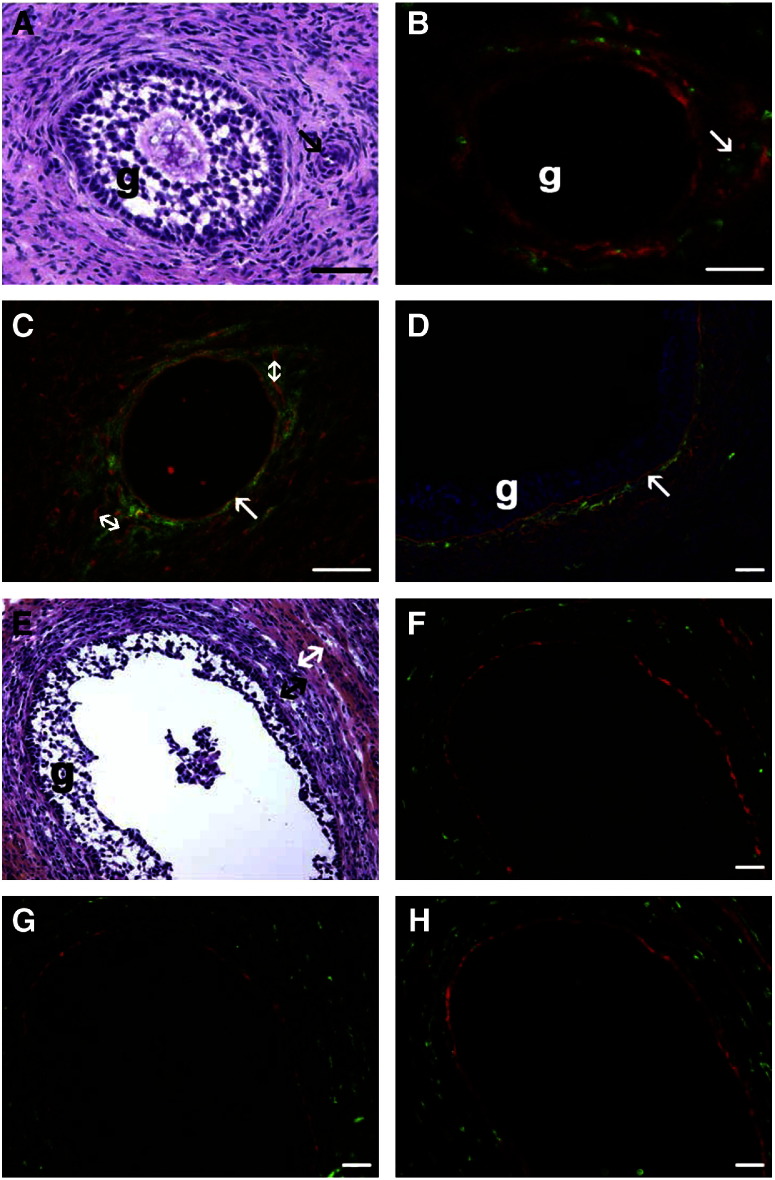
Localization of 3C5, 4C3 and 7D4 CS epitopes in early and small antral follicles. (A) Early antral follicle stained with H&E (g = membrana granulosa, arrow = arteriole). (B) Same follicle as shown in panel A with 3C5 (red) localized to the stromal connective tissue around the follicle and von Willebrand factor indicating blood vessels (green) (arrow = arteriole). (C, D) Combined immunostaining of 7D4 (green) and laminin 111 (red) of an early antral follicle (panel C) and a small antral follicle (panel D). 7D4 is localized to the stromal connective tissue surrounding the early antral follicle (panel C) and laminin 111 is localized to the follicular basal lamina (arrow) and capillary sub-endothelial basal laminas (double headed arrows). In the small antral follicle (panel D) 7D4 (green) is localized to the theca interna (g = membrana granulosa, arrow = follicular basal lamina, DAPI staining of nuclei in blue). (E) Small antral follicle stained with H&E. The specialized connective tissue layers surrounding antral follicles consist of the theca interna (black double-headed arrow) and theca externa (white double-headed arrow) (g = membrana granulosa). (F, G, H) Same follicle as shown in E. 3C5 (red, panel F), 4C3 (red, panel G), 7D4 (red, panel H) localize to the theca interna adjacent to the follicular basal lamina and blood vessels are identified by localization of von Willibrand factor (green). Bars = 20 μm.

**Fig. 3:**
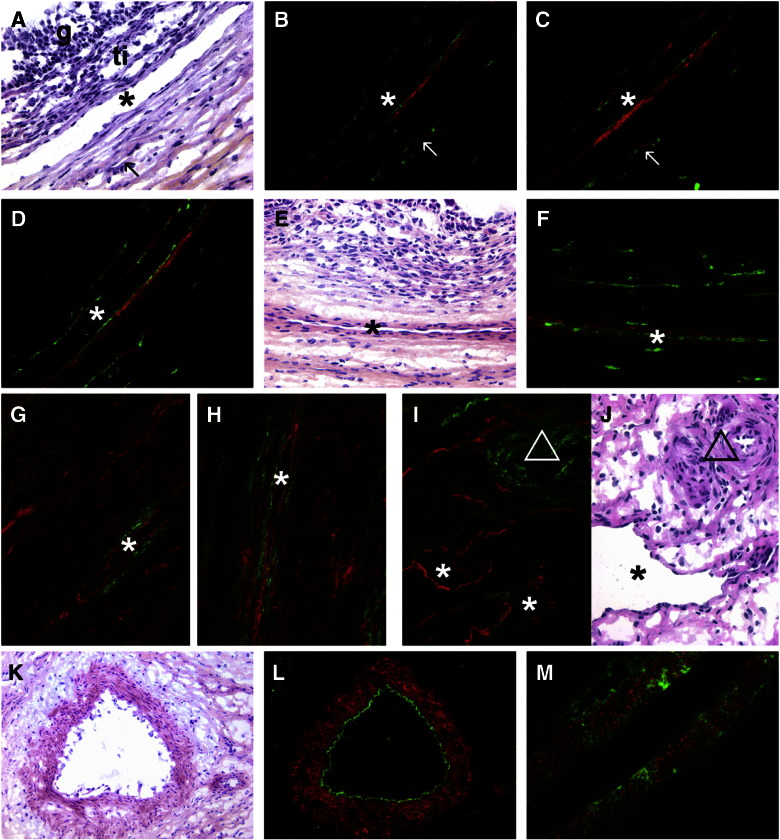
Localization of 3C5, 4C3, 7D4 and 3B3(+) CS epitopes to vessels in the theca externa of large antral follicles and the ovarian medulla. (A) Section of a large antral follicle stained with H&E showing a large vessel (asterisk) at the boundary of the theca interna (ti) and theca externa, the membrana granulosa (g) and a small vessel in the theca externa (arrow). (B, C, D) Serial sections of the area shown in panel A. 3C5 (red, panel B), 4C3 (red, panel C) and 7D4 (red, panel D) localize to the vessels identified by staining with von Willebrand factor (green). The arrow indicates a small vessel in the theca externa and the asterisk a large blood vessel as also identified in panel A. (E) Section of a large antral follicle stained with H&E showing a large vessel (asterisk) in the theca externa. (F) Same section as panel E localizing 3B3(+) (red) and von Willebrand factor (green) around a large blood vessel (asterisk) as also identified in panel E. (G, H, I) Localization of LYVE-1 (red) and of 4C3 (green in G), 7D4 (green in H, I) in the theca externa of large antral follicles (G, H) and ovarian medulla (I). (J) Section of a similar region of the ovarian medulla to that shown in panel I stained with H&E and identifying a lymphatic vessel (asterisk) and an area with aterioles (triangle). (K) A large arteriole in the ovarian medulla stained with H&E. ( L) Serial section of the arteriole in panel K localizing 3B3(+) (red) to the muscularis layer and von Willebrand factor (green) identifying the endothelium. (M) 7D4 (green) localization to the muscularis (red, smooth muscle actin) of a large arteriole in the ovarian medulla. Bars = 20 μm.

**Fig. 4:**
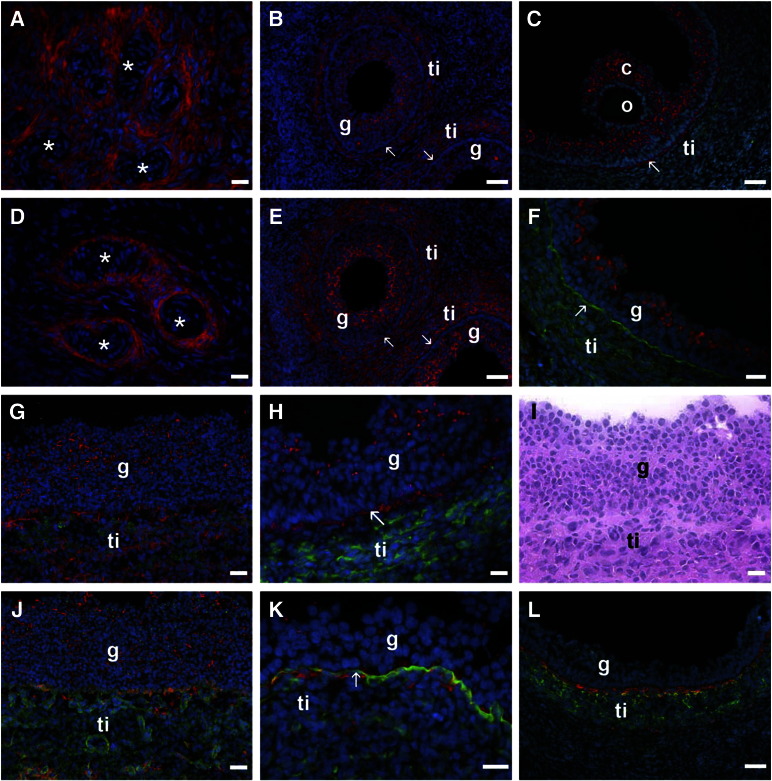
Immunolocalization of CS epitope recognized by 2B6 (red in A-C, G, H) and versican (red in D-F, J, K) in stroma (A, D), early antral follicles (B, E) and antral follicles (C, F, G, H, J-L). CS epitope recognized by 2B6 (A) and versican (D) are localized to the connective tissue stroma surrounding arterioles (asterisks) in the ovarian medulla. In early antral follicles CS epitope 2B6 (B) and versican (E) are localized to the membrana granulosa (g) and theca interna (ti). In antral follicles CS epitope recognized by 2B6 (C, G, H) and versican (F, J, K) are localized to the membrana granulosa (g), the cumulus cells (c) and not the oocyte (O). CS epitope 2B6 (G) and versican (J) are also localized to the theca interna (ti) and form a layer abutting the follicular basal lamina (CS epitope 2B6: C, H; and versican: K). CYP17, a marker of thecal cells, is localized to the theca interna (green in G and H) and laminin (green in J and K) is localized to the follicular basal lamina (arrow) and capillary sub-endothelial basal laminas in the theca interna (ti). Inter-α-trypsin inhibitor (green in L) localizes to the theca interna but does not co-localise with 7D4 (red). (I), H&E, serial section to (G) and (J). Bars = 20 μm (A, D, F-K) and 50 μm (B, C, E, L).

To best illustrate the localization of CS epitopes, versican and inter-α-trypsin inhibitor members, combined immunostaining was conducted to additionally identify laminin 111 in the follicular and sub-endothelial basal laminas (van Wezel et al., 1998), CYP17 which is an enzyme involved in androgen synthesis and within the ovary is specific to steroidogenic cells located within the theca interna (Rodgers et al., 1986), von Willebrand factor in endothelial cells, smooth muscle actin in smooth muscle cells of arterioles and LYVE-1 on the lymphatic vessels located in the theca externa. Additionally nuclei were counter-stained with DAPI and adjacent sections were stained with hematoxylin and eosin (H&E). Antibodies 3C5, 4C3, and 7D4 to CS epitopes localized to the stromal connective tissue surrounding early antral follicles (Fig. 2B,C) and in the theca interna adjacent to the follicular basal lamina in antral follicles (Fig. 2D,F-H). These CS epitopes were also localized to stromal cells surrounding vessels in the theca externa of antral follicles (Fig. 3), as well as vessels in the ovarian medulla, as shown by combined staining with an antibody to von Willebrand factor. Some of these vessels were identified as lymphatics by dual staining with an antibody to LYVE-1 (Fig. 3G, H, I). Antibodies 3C5, 4C3, 7D4 and 3B3(+) did not localize to the capillary plexus within the theca interna of follicles (Fig. 3A-F). 3B3(+) (Fig. 3L) and 7D4 (Fig. 3M), but not 4C3 or 3C5, also localized to the muscularis layer of arterioles. Inter-α-trypsin inhibitor was not localized in any larger blood vessels around follicles where staining with 3C5, 4C3, 7D4 and 3B3(+) was observed, however, it was present in the thecal layer adjacent to the follicular basal lamina (Fig. 4L). No staining was observed with 3B3(−).

The localization pattern of 2B6 was similar to that of versican. Both of the antibodies localized to the stroma surrounding large blood vessels in the ovarian medulla (Fig. 4A, D). In early antral follicles CS epitope 2B6 (Fig. 4B) and versican (Fig. 4E) were localized to the membrane granulosa and theca interna. In antral follicles CS epitope recognized by 2B6 (Fig. 4C, G, H) and versican (Fig. 4F, J, K) were localized to the membrana granulosa preferentially in the apically-situated granulosa cells. CS epitope 2B6 (Fig. 4C, G, H) and versican (Fig. 4J, K) are also localized to the theca interna, sometimes in a layer abutting the follicular basal lamina. 2B6 epitope (Fig. 4C) and versican were also localized to the cumulus cells.

## 3 Discussion

Here we present the first fluorophore-assisted carbohydrate electrophoresis analysis of ovarian follicles at differing developmental stages. We analyzed the two major layers of follicles separately and compared follicles of different sizes as well as healthy and atretic follicles. We discuss these results in conjunction with localization of known PGs. We also conducted further localization of CS PGs some of which localize to groups of stromal cells surrounding some large vessels, including lymphatic vessels, in the theca externa. Based upon findings of others, these cells may therefore represent a stem cell niche as discussed below.

The major disaccharides in follicles derived from CS were ∆Di4S, ∆Di6S and ∆Di0S in thecal tissue and ∆Di4S, ∆Di6S in granulosa cells. The levels were higher in the thecal tissues than granulosa cells. In granulosa cells ∆Di4S was significantly elevated in medium and large follicles and atretic follicles. Atretic follicles also had significantly elevated levels of ∆Di4,6S in granulosa cells. Immunostaining of antral follicles with antibody 2B6 identified 4-sulfated *N*-acetylgalactosamine in theca interna and externa, the membrana granulosa and cumulus cells. In some follicles the localization was preferentially in the apically-situated granulosa cells. Antibodies to 3B3(+) localized 6-sulfated *N*-acetylgalactosamine to the muscularis layer of arterioles; 7D4 also localized to this area. Immunostaining with 7D4, 3C5 and 4C3 which identify epitopes within native CS and DS, localized to the stromal connective tissue surrounding early antral follicles and in the theca interna adjacent to the follicular basal lamina in antral follicles. These CS epitopes were also localized to stromal cells surrounding some vessels in the theca externa of antral follicles as well as vessels in the ovarian medulla. Thus in antral follicles since 3B3(+), 7D4, 3C5 and 4C3 localized to areas different to that observed with 2B6, it would appear that there are at least two CS PGs present in antral follicles or at least one PG but with different CS sulfation pattern. The PG or PG sulfation pattern is predominantly one rich in 4-sulfated *N*-acetylgalactosamine with widespread localization and identified by 2B6. The others patterns have a restricted localization recognised by 3B3(+), 7D4, 3C5 and 4C3 and are likely unsulfated or 6-sulfated *N*-acetylgalactosamine.

As discussed earlier the CS PGs previously identified in ovaries include decorin, versican and bikunin as a component of inter-α-trypsin inhibitor family members. Decorin and versican were identified in a study using small bovine antral follicles (McArthur et al., 2000), however, 4-sulfated N-acetylgalactosamine immunoreactivity in some column chromatography fractions could not be ascribed to the PGs identified in that study, suggesting that there maybe have been a larger PG still to be identified. Since the immunostaining patterns observed here of both versican and 2B6 were similar this would indicate that another 4-sulfated N-acetylgalactosamine PG is not present. The larger unidentified proteoglycan (McArthur et al., 2000) may have been another isoform of versican, and more recent studies have identified two isoforms of versican, V0 and V1, in bovine follicular fluid (Clarke et al., 2006). Hence we now suggest that there is no other larger 4-sulfated N-acetylgalactosamine proteoglycan in bovine antral follicles. We therefore conclude that versican contains 4-sulfated *N*-acetylgalactosamine and that it is the predominant 4-sulfated *N*-acetylgalactosamine containing PG in antral follicles.

Follicles also contain another CS PG with a restricted localization, as observed by staining with antibodies 7D4, 3C5, 4C3 and 3B3(+). Previously it was shown that decorin localizes to the thecal layers of bovine antral follicles and is uniformly distributed within these layers, more strongly in the theca externa than interna (Irving-Rodgers and Rodgers, 2007). Bikunin has not been localized specifically, but as a component of serum it should preferentially be present in capillaries of theca. In mouse ovaries (Irving-Rodgers and Rodgers, 2005), and as shown here in the bovine, components of inter-α-trypsin inhibitor localize to the theca interna and also the membrana granulosa in large antral follicles. Additionally, inter-α-trypsin inhibitor has a low 4-sulfated CS side chain (Kakizaki et al., 2007). None of the staining patterns observed here with 7D4, 3C5, 4C3 or 3B3(+) resembled the localization pattern of decorin or inter-α-trypsin inhibitor members. This suggests that another CS PG(s) could be present in bovine antral follicles and in the theca externa associated with cells around larger blood vessels in particular. Based upon the fluorophore-assisted carbohydrate electrophoresis analysis the CS PG(s) is likely to be unsulfated or 6-sulfated *N*-acetylgalactosamine. The pattern of immunostaining suggests that the PG(s) are not associated with cell surfaces and therefore the PG(s) is unlikely to be any of the transmembrane PG such as the syndecans or CSPG4 (Couchman, 2010). Epitopes for 7D4, 3C5, 4C3 or 3B3(+) have been examined in a variety of tissues including the intervertebral disc (Hayes et al., 2001, 2011) and articular cartilage (Hayes et al., 2008b). In the former, these epitopes localize to regions rich in stem cells, suggesting that they could contribute to the stem cell niche (Hayes et al., 2008b). Expression of these epitopes in vertebral disc is especially interesting as the pattern of each changes during growth, development and ageing (Hayes et al., 2011). The identity of these PGs in the follicle is unknown but their localization suggests that this group of cells whose identity is unknown at this stage may have a unique role. It is possible that they are progenitor cells. Evidence for somatic stem cells has been shown previously both for the theca (Honda et al., 2007) and granulosa cells (Lavranos et al., 1994, 1996, 1999; Rodgers et al., 1999), so the existence of a perivascular progenitor cell population within the follicle is not unexpected but not previously identified.

The level of HA was unchanged in healthy and atretic follicles and in follicles of different sizes in either granulosa cells or thecal tissues. The HA localizes to cumulus cells, antrally-situated granulosa cells and to the stromal side of the follicular basal lamina in bovine follicles (Irving-Rodgers and Rodgers, 2005). Its production by cumulus cells increases dramatically after the surge release of luteinizing hormone in ovulating follicles (Salustri et al., 1992, 1999) and primarily this is via hyaluronan synthase (HAS) 2 (Ochsner et al., 2003; Schoenfelder and Einspanier, 2003). The follicles examined in this study were not ovulatory and the levels of HA would not have been expected to change dramatically. The enzyme responsible for the polymerisation of hyaluronan in the follicle before ovulation is HAS1, at least in pigs. It is expressed at a higher level in the theca of porcine follicles in comparison to granulosa cells, but is upregulated in granulosa cells late in atresia while hyaluronan levels also increased late in atresia (Miyake et al., 2009).

Of the saccharides derived from HS, ΔU-G-NAc, ΔU-G-NS, ΔU(2S)-G-NS and ΔU(2S)-G(6S)-NS were all significantly elevated in thecal tissue compared to granulosa cells and the predominant ones in order were ΔU-G-NAc, ΔU-G-NS and ΔU(2S)-G(6S)-NS. In granulosa cells the levels of ΔU-G-NAc and ΔU-G-NS were significantly elevated in medium and large-sized follicles and in atretic follicles. In thecal tissue the levels of ΔU-G-NAc, ΔU-G(6S)-NAc, ΔU-G-(6S)-NS, ΔU(2S)-G-NS and ΔU(2S)-G(6S)-NS were significantly elevated in large follicles and the levels of ΔU-G-NAc, ΔU-G-(6S)-NS, and ΔU(2S)-G-NS were significantly elevated in atretic follicles. The better known HS PGs in follicles are perlecan, collagen type XVIII, betaglycan and anticoagulant HSPGs. Perlecan and collagen XVIII are present in the follicular basal lamina and sub-endothelial basal laminas in the theca layers of antral bovine follicles (McArthur et al., 2000; Irving-Rodgers and Rodgers, 2006). They are also present in focimatrix which increases in amount as follicles enlarge (Irving-Rodgers et al., 2004; Irving-Rodgers and Rodgers, 2006). During atresia when the follicular cells are dying these basal lamina components are not degraded (McArthur et al., 2000), unlike at ovulation (Irving-Rodgers et al., 2006). This may explain in part why HS-derived disaccharides increased in atretic follicles as the results are on a per DNA basis. Betaglycan in bovine antral follicles is significantly higher in the theca than in granulosa cells and positively correlated with increasing follicles size, at least in the thecal layer (Glister et al., 2010). HS PGs containing the antithrombin-binding pentasaccharide of heparin are located in the endothelial cells of the thecal layer and in granulosa cells as described in rat ovaries (Hasan et al., 2002; de Agostini et al., 2008). Other HS PGs in follicles include syndecans, glypicans and CD44. The information of the former two is very limited. CD44 is first detected in infiltrating macrophages as atresia progresses in procine follicles but this CD44 is not heparan sulfated (Miyake et al., 2006). Hence CD44 is unlikely to be a source of any HS disaccharides as observed by our analyses. The concentrations of many of the HS-derived saccharides in both thecal layers and the membrana granulosa correlated with each other suggesting that the HS side-chains were uniform in composition.

The quantity of a number of the disaccharides were correlated with each other. This would be expected to some degree within each class of disaccharides derived from CS, HA or HS if there were relatively few PGs and if their glycosaminoglycans were relatively constant in composition during follicular growth or atresia, as was largely the case. Of potential significance are the correlations between the CS-derived ∆Di4S and the HS-derived ΔU-G-NAc, ΔU-G-NS, ΔU-G-(6S)-NS, ΔU(2S)-G-NS and ΔU(2S)-G(6S)-NS in granulosa cells. As discussed above ∆Di4S is probably derived from versican and the HS disaccharides from perlecan and collagen XVIII found in focimatrix or betaglycan. However, versican does not co-localize with focimatrix (Irving-Rodgers et al., 2004), despite being in greater abundance amongst the antrally-situated granulosa cells as is focimatrix (Irving-Rodgers et al., 2004). In the theca, versican is adjacent to the follicular basal lamina but is not part of it (Irving-Rodgers et al., 2006). Thus there clearly is a hitherto unrecognized relationship between versican and betaglycan or the basal lamina components of the granulosa cell compartments. The significance of this relationship is not known.

In summary, we identified the major disaccharides in thecal layers and the membrana granulosa as CS-derived ∆Di4S and ∆Di6S and the HS-derived ΔU-G-NAc, with elevated levels in the thecal layers. Increasing size and atresia lead to increased levels of some of the disaccharides. The effect of size appears at odds with earlier research showing decreasing levels in the follicular fluid with increasing size (Grimek and Ax, 1982), however, the levels examined here were those in the cellular thecal and granulosa layers, not the follicular fluid. We conclude that versican is 4-sulfated *N*-acetylgalactosamine and is the predominant 4-sulfated *N*-acetylgalactosamine containing PG in antral follicles. CS PG in follicular fluid identified as containing versican has been shown to be osmotic and is proposed to be involved in formation of follicular fluid (Clarke et al., 2006; Rodgers and Irving-Rodgers, 2010a). Another unsulfated or 6-sulfated *N*-acetylgalactosamine PG, which is not decorin or a member of inter-α-trypsin inhibitor family, could be present in bovine antral follicles located around the larger blood vessels in the theca externa and associated with a group of cells whose identity is unknown at this stage. The sulfation patterns of HS PGs appear uniform and the HS is probably associated with basal lamina components, perlecan and collagen XVIII, or betaglycan or possibly with the poorly characterised cell surface PGs. Collectively these studies show clearly that CS PGs are dynamic during follicular growth and atresia and probably have a variety of roles in these processes.

## 4 Experimental procedures

### 4.1 Tissues

For fluorophore-assisted carbohydrate electrophoresis analyses, ovaries were collected at an abattoir from *Bos taurus* cows, visually assessed as non-pregnant, and transported to the laboratory on ice in Hank's balanced-salt solution (HBSS) without calcium or magnesium (H 2387; Sigma Bio Science, St. Louis, MO). The external diameter of follicles was measured with callipers and then the follicles were cut open and a portion through each follicle wall (approximately 2 × 2 × 2 mm) was fixed in 2.5% glutaraldehyde in 0.1 M phosphate buffer. The remainder of the follicle was removed to HBSS and granulosa cells scraped from the inside of the follicle. Granulosa cells were washed with 1 ml of HBSS by centrifugation for 5 min at 3,000 g and the supernatant was removed. The inside of the follicle was rinsed with HBSS to remove any remaining granulosa cells and the thecal tissue was dissected away from the ovarian stroma. This tissue is mostly interna with some components of externa (unpublished observations) but in the literature the tissue derived from this method is referred to as theca interna. Fig. 2E illustrates the location of granulosa cells and both thecal layers in an antral follicle. Both follicle components were snap frozen on dry ice and stored at − 20 °C for subsequent fluorophore-assisted carbohydrate electrophoresis analyses. For immunohistochemistry whole ovaries were collected from the same abattoir and frozen in OCT compound and stored at − 80 °C.

### 4.2 Histological classification of follicles

For light microscopy of glutaraldehyde-fixed follicle wall, specimens were post-fixed in 1% osmium tetroxide and embedded in epoxy resin as previously described (Irving-Rodgers et al., 2002). Sections of 0.5 μm in thickness were cut with glass knives using a Richert-Jung Ultracut E ultramicrotome (Leica Microsystems Pty Ltd, North Ryde, NSW, Australia). Sections were fixed onto plain glass slides by drying at 90 °C and stained with 1% aqueous methylene blue in 1% sodium tetraborate (ProSciTech, Thuringowa, QLD, Australia). The membrana granulosa was observed by light microscopy for classification of follicles as healthy or atretic (Irving-Rodgers et al., 2009; Rodgers and Irving-Rodgers, 2010b). Follicles were assessed as healthy or atretic based upon the morphology of the membrana granulosa and the presence or absence of dead cells, as previously described (Irving-Rodgers et al., 2001, 2003).

### 4.3 Fluorophore-assisted carbohydrate electrophoresis analysis

Thecal and granulosa cells from six healthy and six atretic follicles in each of three size ranges: small (3–5 mm), medium (6-9 mm) and large (10 mm or more) from 29 animals were selected for analyses. Samples were analysed as reported previously (Nigro et al., 2010), with slight modifications. Granulosa or theca cells were treated with 0.2 mg/ml proteinase K (Invitrogen Australia Pty. Ltd., Mt Waverley, VIC, Australia) containing sodium dodecyl sulfate (0.01% w/v) in a final volume of 250 μl. Samples were incubated at 60 °C for 4 h, with regular vortexing and brief centrifugation every hour. A small aliquot (20 μl) of each digest was removed for DNA analysis using the Quant-iT Pico Green dsDNA Assay Kit (Invitrogen Australia Pty. Ltd) according to the manufacturer's instructions. The concentration of DNA in each sample was used to determine the volume of each sample in order to load equivalent amounts of DNA into each well in electrophoretic gels. The remainder of the samples were treated with 4 volumes of 100% ethanol to precipitate glycosaminoglycans.

The ethanol-precipitated glycosaminoglycans were first resuspended in 40 μl of 0.1 M ammonium acetate pH 7.0/0.025% bovine serum albumin (BSA) containing 25 mU/ml hyaluronidase SD (Seikagaku Corporation, Tokyo, Japan) and 250 mU/ml chondroitinase ABC (Seikagaku Corporation, Tokyo, Japan) and digested for 3 h at 37 °C. The remaining undigested glycosaminoglycans were precipitated with 4 volumes of 100% ethanol and the supernatant containing the digested products of the hyaluronidase and chondroitinase ABC was lyophilized and subsequently labelled with 6.25 mM 2-aminoacridone/0.625 mM sodium cyanoborohydride/7.5% (v/v) acetic acid for 16 h at 37 °C. For analysis of these digested products, fluorescently-tagged disaccharides and C-Kit standards (Seikagaku, Tokyo, Japan) were separated on gels containing 20% (v/v) acrylamide/bis with 4 mM Tris acetate, pH 7.0, and 2.5% (v/v) glycerol using a constant current (15 mA/gel) at 4 °C in TBE buffer.

Following digestion with chondroitinase and hyaluronidase and removal of the digested products, the remaining undigested ethanol-precipitated glycosaminoglycans were treated with a cocktail of heparinase and heparitinase I and II (each at 28 mU/ml; Seikagaku Corporation, Tokyo, Japan) in 20 μl of 0.1 M ammonium acetate pH 7.0/0.2% BSA for 4 h at 37 °C. After digestion the disaccharides were lyophilized and labeled with the 6.25 mM 2-aminoacridone/0.625 mM sodium cyanoborohydride solution containing 0.75% (v/v) acetic acid. For analysis of these products, the disaccharides and H-Kit standards were separated on N-linked oligosaccharide profiling gels (Prozyme, Hayward, CA, USA) with a constant current (20 mA/gel) at 4 °C in the commercial buffer provided.

In each gel one sample from each of the six types of follicle (healthy or atretic of the three different sizes) was included from either of the follicle layers (thecal layers or granulosa cells). In each gel dissacharide standards (Std) and a control, in which buffer replaced a sample before reaction with 2-aminoacridone (Blank), were also included. An equivalent amount of each sample containing 3 μg of DNA was loaded per lane. Images of gels were captured with a cooled CCD camera associated with the LAS-3000 imager (Fujifilm Corp., Tokyo, Japan). Multi Gauge Version 3.0 software (Fujifilm Corp.) was used to quantitate the intensity of the saccharide bands in the gels.

### 4.4 Immunohistochemistry

Portions of whole ovaries (n = 14) embedded in OCT compound were used for localization using an indirect immunofluorescence method. Tissue sections (5 μm) were cut from each of the frozen ovaries using a CM1800 Leica cryostat (Leica Microsystems Pty. Ltd., North Ryde, NSW, Australia), collected on Superfrost glass slides (HD Scientific Supplies, Australia) and stored at − 20°C until use. Sections were dried under vacuum for 5 min and in some cases, as indicated below, were incubated with chondroitinase ABC lyase (C-3667, Sigma Chemical Co.) at a concentration of 0.05 units/ml in 0.1 M Tris acetate buffer, pH8.0 for 1 h at 37 °C. In some cases the sections were fixed in 10% neutral buffered formalin for 5 min (when using antibodies 2B6, 12C5 and 7D4 when in combination with anti inter-α-trypsin inhibitor). Sections were rinsed three times for 5 min in hypertonic phosphate-buffered saline (hPBS, 10 mM sodium/potassium phosphate with 0.274 M NaCl, 5 mM KCl pH 7.2) before treatment with blocking solution [10% normal donkey serum (D-9663, Sigma Chemical Co.) in antibody diluent containing 0.55 M NaCl and 10 mM sodium phosphate (pH 7.1)] for 20 min at room temperature. Primary antibodies directed against CS/DS epitopes were 2B6 [murine monoclonal antibody 2B6, IgG purified from ascites fluid, recognizes a disaccharide containing a non-reducing 4,5 unsaturated hexuronate adjacent to a 4-sulfated *N*-acetylgalactosamine which is produced by chondroitinase digestion of native CS or DS chains; 1:1000 dilution; (Caterson et al., 1985, 1987; Hayes et al., 2008a)], 3B3 [murine monoclonal antibody 3B3, IgM purified from ascites fluid, recognizes a disaccharide epitope containing a non-reducing unsaturated hexuronate adjacent to a 6-sulfated *N*-acetylgalactosamine which is produced by chondroitinase digestion [designated 3B3(+)], or without chondroitinase digestion 3B3 recognizes a native epitope on CS chains containing a non-reducing unsaturated or saturated hexuronate adjacent to a 6-sulfated *N*-acetylgalactosamine [designated 3B3(−)]; 1:1000 dilution; (Caterson et al., 1985, 1987; Hayes et al., 2008a)], 7D4 [murine monoclonal antibody IgM, recognizes an epitope within native CS/DS; 1:1000 dilution; (Hayes et al., 2008a)], 3C5 [murine monoclonal antibody IgG recognizes an epitope within native CS/DS; 1:1000 dilution (Caterson et al., 1985)] and 4C3 [murine monoclonal antibody IgM, recognizes an epitope within native CS/DS; 1:1000 dilution, (Hayes et al., 2008a)]. . Immunostaining for CS or CS PGs was conducted alone or in combination with rabbit anti-human von Willebrand factor (Catalogue # 0082, Dako Australia Pty Ltd at a concentration of 7 μg/ml), rabbit anti-human alpha smooth muscle actin (Abcam, Cat # ab5694 used at 2 μg/ml), rabbit anti- mouse LYVE-1 (Upstate/ Millipore, Catalogue # 07–538, used at 0.5 μg/ml) rabbit anti-human CYP17 [1:1000; (Conley et al., 1995)] and rabbit anti-mouse laminin 111 (Sigma, Catalogue # L9393, 1:100 dilution) to identify different regions of the ovary. Human versican [12C5; 1:100 dilution; Developmental Studies Hybridoma Bank, Iowa City, IA (Sztrolovics et al., 2002)] and inter-α-trypsin inhibitor (Dako, #A0301, rabbit anti-human, at a concentration of 15.2 μg/ml) were also localized. Negative controls included no primary antisera and non-immune mouse serum. No staining of ovaries was observed with these controls.

All secondary antibodies and conjugated fluorophores were purchased from Jackson ImmunoResearch Laboratories Inc. (West Grove, PA, USA). Secondary antibodies used were biotin-SP-conjugated AffiniPure donkey anti-mouse IgG (1:100; Cat. # 715-065-020) followed by Cy3-conjugated streptavidin (1:100; Cat. # 016-160-084) and donkey anti-rabbit IgG conjugated to FITC (1:100; Cat. # 712-1096-153), or biotin-SP-conjugated AffiniPure donkey anti-mouse IgG followed by DTAF-conjugated streptavidin (1:100; Catalogue # 016-010-084) and donkey anti-rabbit IgG conjugated to Cy3 (1:100; Catalogue # 711-166-152) Sections were also treated with the nuclear stain 4’,6’-diamidino-2-phenylindole dihydrochloride (DAPI) solution (Molecular Probes, Eugene, OR, USA) and coverslips were attached with medium for fluorescence (Cat. # S3023; Dako Corporation, Carpinteria, CA, USA). Sections were photographed with an Olympus BX50 microscope with an epifluorescence attachment and a Spot RT digital camera (Diagnostic Instruments Inc., Sterling Heights, MI, USA).

### 4.5 Statistical analyses

All calculations were performed using SAS Version 9.2 (SAS Institute Inc., Cary, NC, USA). Some saccharides were not normally distributed due to multiple values falling below the limit of detection. Since these values were not able to be transformed to achieve a normal distribution non-parametric analyses were conducted. In these cases a Wilcoxon's signed rank test, the non-parametric equivalent of a paired Student's *t* test, or a Kruskal Wallis test, the non-parametric equivalent of a one-way ANOVA, were carried out to compare saccharide concentrations between granulosa and thecal cells, and for each cell type between follicles of different sizes and between healthy and atretic follicles. Similarly for correlation analyses the non-parametric Spearman's correlation coefficients were calculated.

## Figures and Tables

**Table 1:** Comparison of the saccharide concentrations (pmoles per μg DNA) of theca cells versus granulosa cells combining all follicle types examined.

	Saccharides	Abbreviations	Cell type				*p-value
			Granulosa		Theca		
			Median	Inter-quartile Range	Median	Inter-quartile Range	
Chondroitin saccharides	∆uronic acid with *N* -acetylgalactosamine	∆Di0S	0.0	0.0	5.2	7.0	0.001
	∆uronic acid with *4* –sulfated *N*-acetylgalactosamine	∆Di4S	5.4	7.8	39.0	31.2	0.021
	∆uronic acid with *6* -sulfated *N* –acetylgalactosamine	∆Di6S	6.6	6.9	20.1	9.6	0.001
	∆uronic acid with *4* and *6* sulfated *N* –acetylgalactosamine	∆Di4,6S	0.0	0.0	0.0	0.0	0.375
	*2*-sulfated ∆uronic acid with *4-*and *6-*sulfated *N* –acetylgalactosamine	∆Di2,4,6S	0.0	0.0	0.0	0.0	0.500
Hyaluronan saccharide	∆uronic acid with *N* -acetylglucosamine (beta 1–4 hydroxy linkage)	∆DiHA	0.0	0.5	4.8	9.7	0.219
† Heparan saccharides	∆uronic acid with *N* -acetylglucosamine (beta 1–3 hydroxy linkage)	ΔU-G-NAc	1.3	1.8	2.5	2.0	<.001
	∆uronic acid with *N* sulfated glucosamine	ΔU-G-NS	0.0	0.1	0.6	1.2	<.001
	∆uronic acid with *6-*sulfated *N* -acetylglucosamine	ΔU-G(6S)-NAc	0.0	0.0	0.0	0.0	0.570
	∆uronic acid with *N-*and *6-*sulfated acetylglucosamine	ΔU-G-(6S)-NS	0.0	0.0	0.0	0.4	0.055
	*2*-sulfated ∆uronic acid with *N-*sulfated acetylglucosamine	ΔU(2S)-G-NS	0.0	0.0	0.0	0.4	0.006
	*2*-sulfated ∆uronic acid with *N-*and *6-*sulfated acetylglucosamine	ΔU(2S)-G(6S)-NS	0.0	0.0	0.4	0.6	< 0.001

*Wilcoxon signed rank test. † Number of observations is n = 24 except for the HS saccharides where n = 36.

**Table 2:** Comparison of the saccharide concentrations (pmoles per μg DNA) in granulosa cells from small, medium and large-sized follicles.

	Saccharides	Follicle Size						
		Small		Medium		Large		*p-value
		Median	Inter-quartile Range	Median	Inter-quartile Range	Median	Inter-quartile Range	
Chondroitin saccharides	∆Di0S	0.0	0.0	0.0	0.0	0.0	0.0	1.000
	∆Di4S	1.5	4.3	5.7	6.6	7.5	24.3	0.019
	∆Di6S	5.9	8.1	6.2	7.3	7.2	5.6	0.992
	∆Di4,6S	0.0	0.0	0.0	0.0	0.0	2.8	0.109
	∆Di2,4,6S	0.0	0.0	0.0	0.0	0.0	0.0	1.000
Hyaluronan saccharide	∆DiHA	0.0	0.0	0.0	0.5	0.0	2.6	0.398
† Heparan saccharides	ΔU-G-NAc	0.0	1.6	1.8	1.7	1.2	0.3	0.027
	ΔU-G-NS	0.0	0.0	0.1	0.5	0.0	0.1	0.017
	ΔU-G(6S)-NAc	0.0	0.0	0.0	0.4	0.0	0.0	0.061
	ΔU-G-(6S)-NS	0.0	0.0	0.0	0.5	0.0	0.0	0.075
	ΔU(2S)-G-NS	0.0	0.0	0.0	0.1	0.0	0.0	0.858
	ΔU(2S)-G(6S)-NS	0.0	0.0	0.0	0.2	0.0	0.0	0.550

* Kruskal Wallis test. † Number of observations is n = 8 except for HS saccharides where n = 12.

**Table 3:** Comparison of the saccharide concentrations (pmoles per μg DNA) in granulosa cells from healthy and atretic follicles.

	Saccharides	Health				
		Healthy		Atretic		*p-value
		Median	Inter-quartile Range	Median	Inter-quartile Range	
Chondroitin saccharides	∆Di0S	0.0	0.0	0.0	0.0	1.000
	∆Di4S	4.1	3.8	10.7	24.5	0.022
	∆Di6S	5.8	5.6	7.7	7.5	0.207
	∆Di4,6S	0.0	0.0	0.0	1.7	0.048
	∆Di2,4,6S	0.0	0.0	0.0	0.0	1.000
Hyaluronan saccharide	∆DiHA	0.0	0.0	0.0	2.1	0.082
† Heparan saccharides	ΔU-G-NAc	0.0	1.2	1.6	1.0	0.001
	ΔU-G-NS	0.0	0.0	0.0	0.4	0.061
	ΔU-G(6S)-NAc	0.0	0.0	0.0	0.3	0.025
	ΔU-G-(6S)-NS	0.0	0.0	0.0	0.1	0.080
	ΔU(2S)-G-NS	0.0	0.0	0.0	0.1	0.251
	ΔU(2S)-G(6S)-NS	0.0	0.0	0.0	0.3	0.013

*Wilcoxon test. † Number of observations is n = 12 except for the HS saccharides where n = 18.

**Table 4:** Comparison of the saccharide concentrations (pmoles per μg DNA) in thecal cells from small, medium and large-sized follicles.

	Saccharides	Follicle Size						
		Small		Medium		Large		*p-value
		Median	Inter-quartile Range	Median	Inter-quartile Range	Median	Inter-quartile Range	
Chondroitin saccharides	∆Di0S	5.3	4.0	1.4	5.4	7.3	9.8	0.161
	∆Di4S	39.0	27.6	31.8	30.3	47.0	23.4	0.213
	∆Di6S	23.6	11.7	15.9	6.3	21.4	17.5	0.033
	∆Di4,6S	0.0	0.0	0.0	0.0	0.0	0.8	0.742
	∆Di2,4,6S	0.0	0.0	0.0	0.0	0.0	0.0	0.593
Hyaluronan saccharide	∆DiHA	5.5	5.1	0.0	7.4	3.8	12.8	0.522
† Heparan saccharides	ΔU-G-NAc	2.3	1.8	1.9	1.0	3.7	1.2	0.001
	ΔU-G-NS	0.4	1.3	0.2	0.7	0.8	2.0	0.236
	ΔU-G(6S)-NAc	0.0	0.0	0.0	0.0	0.0	0.7	0.019
	ΔU-G-(6S)-NS	0.1	0.4	0.0	0.0	0.4	0.3	0.002
	ΔU(2S)-G-NS	0.1	0.5	0.0	0.0	0.4	0.5	0.001
	ΔU(2S)-G(6S)-NS	0.0	0.0	0.0	0.0	0.0	0.0	0.001

*Kruskal Wallis test. † Number of observations is n = 8 except for the HS saccharides where n = 12.

**Table 5:** Comparison of the saccharide concentrations (pmoles per μg DNA) in thecal cells from healthy and atretic follicles.

	Saccharides	Health				
		Healthy		Atretic		*p-value
		Median	Inter-quartile Range	Median	Inter-quartile Range	
Chondroitin saccharides	∆Di0S	5.4	7.1	5.0	7.0	0.816
	∆Di4S	38.1	19.7	45.7	38.6	0.323
	∆Di6S	21.4	10.6	18.7	9.7	0.797
	∆Di4,6S	0.0	0.0	0.0	0.5	0.338
	∆Di2,4,6S	0.0	0.0	0.0	0.0	0.179
Hyaluronan saccharide	∆DiHA	2.6	10.6	5.1	9.0	0.700
† Heparan saccharides	ΔU-G-NAc	1.9	1.3	3.0	2.1	0.013
	ΔU-G-NS	0.0	0.9	0.7	2.2	0.054
	ΔU-G(6S)-NAc	0.0	0.0	0.0	0.5	0.072
	ΔU-G-(6S)-NS	0.0	0.2	0.4	0.4	0.045
	ΔU(2S)-G-NS	0.0	0.2	0.2	0.8	0.030
	ΔU(2S)-G(6S)-NS	0.3	0.5	0.5	0.7	0.122

*Wilcoxon test. † Number of observations n = 12 except for the HS saccharides where n = 18.

## Appendix A Supplementary data

Supplemental Table 1 Spearman correlation coefficients between saccharide concentrations using all data from granulosa cells from all follicles examined.

Supplemental Table 2 Spearman correlation coefficients between saccharide concentrations using all data from thecal cells from all follicles examined.

Supplemental Table 3 Spearman correlation coefficients between saccharide concentrations using all data from theca and granulosa cells from all follicles examined.
