# Supplementary material for: Glycomic analyses of ovarian follicles during development and atresia
Source: Matrix Biol. 2012 Jan;31(1):45–56. doi: 10.1016/j.matbio.2011.10.002 (PMC3657699; doi:10.1016/j.matbio.2011.10.002)
Supplement: Supplemental Table 1 — Spearman correlation coefficients between saccharide concentrations using all data from granulosa cells from all follicles examined. [file mmc1.doc]

**Supplemental Table 1**

Spearman correlation coefficients between saccharide concentrations using all data from granulosa cells from all follicles examined.

|  | **Chondroitin saccharides** | | | | | **Hyaluronan saccharide** | **Heparan saccharides** | | | | | |
| --- | --- | --- | --- | --- | --- | --- | --- | --- | --- | --- | --- | --- |
| **Saccharides** | ∆Di-0S | ∆Di-4S | ∆Di6S | ∆Di-4,6S | ∆Di-2,4,6S | ∆Di-HA | ΔU-G-NAc | ΔU-G-NS | ΔU-G(6S)-NAc | ΔU-G-(6S)-NS | ΔU(2S)-G-NS | ΔU(2S)-G(6S)-NS |
| ∆Di0S | . | . | . | . | . | . | . | . | . | . | . | . |
|  | . | . | . | . | . | . | . | . | . | . | . | . |
| ∆Di4S |  | 1.000 | 0.245 | 0.591 | . | 0.413 | 0.520 | 0.490 | 0.328 | 0.404 | 0.493 | 0.507 |
|  |  |  | (0.248) | (0.002) | . | (0.045) | (0.009) | (0.015) | (0.118) | (0.050) | (0.014) | (0.012) |
| ∆Di6S |  |  | 1.000 | 0.328 | . | 0.064 | 0.455 | 0.189 | 0.097 | 0.152 | 0.074 | 0.040 |
|  |  |  |  | (0.118) | . | (0.765) | (0.025) | (0.378) | (0.652) | (0.477) | (0.731) | (0.852) |
| ∆Di4,6S |  |  |  | 1.000 | . | 0.091 | 0.190 | 0.227 | 0.029 | 0.029 | 0.099 | 0.009 |
|  |  |  |  |  | . | (0.672) | (0.375) | (0.286) | (0.892) | (0.892) | (0.647) | (0.965) |
| ∆Di2,4,6S |  |  |  |  | . | . | . | . | . | . | . | . |
|  |  |  |  |  | . | . | . | . | . | . | . | . |
| ∆DiHA |  |  |  |  |  | 1.000 | 0.270 | 0.151 | 0.236 | 0.209 | 0.152 | 0.343 |
|  |  |  |  |  |  |  | (0.202) | (0.483) | (0.267) | (0.328) | (0.479) | (0.100) |
| ΔU-G-NAc |  |  |  |  |  |  | 1.000 | *0.569 | *0.454 | *0.443 | *0.320 | *0.506 |
|  |  |  |  |  |  |  |  | (<0.001) | (0.005) | (0.007) | (0.057) | (0.002) |
| ΔU-G-NS |  |  |  |  |  |  |  | 1.000 | *0.558 | *0.828 | *0.546 | *0.612 |
|  |  |  |  |  |  |  |  |  | (<0.001) | (<0.001) | (0.001) | (<0.001) |
| ΔU-G(6S)-NAc |  |  |  |  |  |  |  |  | 1.000 | *0.520 | *0.402 | *0.305 |
|  |  |  |  |  |  |  |  |  |  | (0.001) | (0.015) | (0.070) |
| ΔU-G-(6S)-NS |  |  |  |  |  |  |  |  |  | 1.000 | *0.371 | *0.617 |
|  |  |  |  |  |  |  |  |  |  |  | (0.026) | (<0.001) |
| ΔU(2S)-G-NS |  |  |  |  |  |  |  |  |  |  | 1.000 | *0.581 |
|  |  |  |  |  |  |  |  |  |  |  |  | (<.0.001) |
| ΔU(2S)-G(6S)-NS |  |  |  |  |  |  |  |  |  |  |  | 1.000 |
|  |  |  |  |  |  |  |  |  |  |  |  |  |

Values in each square are the Spearman correlation coefficient, and below in brackets the Probability > |r| under H0:Rho=0.

The number of observations is n =24 except where *indicates n = 36.

**Supplemental Table 2**

Spearman correlation coefficients between saccharide concentrations using all data from thecal cells from all follicles examined.

|  | **Chondroitin saccharides** | | | | | **Hyaluronan**  **saccharide** | **Heparan saccharides** | | | | | |
| --- | --- | --- | --- | --- | --- | --- | --- | --- | --- | --- | --- | --- |
| **Saccharides** | ∆DiOS | ∆Di4S | ∆Di6S | ∆Di4,6S | ∆Di2,4,6S | ∆DiHA | ΔU-G-NAc | ΔU-G-NS | ΔU-G(6S)-NAc | ΔU-G-(6S)-NS | ΔU(2S)-G-NS | ΔU(2S)-G(6S)-NS |
| ∆DiOS | 1.000 | 0.318 | 0.164 | 0.235 | 0.266 | -0.315 | -0.054 | 0.126 | 0.507 | -0.001 | 0.263 | 0.069 |
|  | . | (0.130) | (0.444) | (0.269) | (0.208) | (0.134) | (0.803) | (0.556) | (0.011) | (0.996) | (0.214) | (0.750) |
| ∆Di4S |  | 1.000 | 0.783 | 0.564 | 0.261 | 0.633 | 0.344 | -0.073 | 0.296 | 0.195 | 0.355 | 0.195 |
|  |  |  | (<0.001) | (0.004) | (0.217) | (0.001) | (0.099) | (0.733) | (0.160) | (0.360) | (0.089) | (0.361) |
| ∆Di6S |  |  | 1.000 | 0.305 | 0.087 | 0.797 | 0.401 | -0.067 | 0.331 | 0.210 | 0.388 | 0.266 |
|  |  |  |  | (0.147) | (0.686) | (<0.001) | (0.052) | (0.755) | (0.114) | (0.325) | (0.061) | (0.209) |
| ∆Di4,6S |  |  |  | 1.000 | 0.604 | 0.460 | 0.204 | -0.031 | 0.501 | 0.004 | 0.189 | 0.222 |
|  |  |  |  |  | (0.002) | (0.024) | (0.340) | (0.884) | (0.013) | (0.984) | (0.376) | (0.297) |
| ∆Di2,4,6S |  |  |  |  | .1.000 | 0.136 | 0.164 | 0.081 | 0.379 | 0.310 | 0.256 | 0.135 |
|  |  |  |  |  |  | (0.527) | (0.444) | (0.705) | (0.068) | (0.141) | (0.226) | (0.530) |
| ∆DiHA |  |  |  |  |  | 1.000 | 0.344 | -0.191 | 0.148 | 0.110 | 0.186 | 0.172 |
|  |  |  |  |  |  |  | (0.100) | (0.372) | (0.491) | (0.608) | (0.385) | (0.423) |
| ΔU-G-NAc |  |  |  |  |  |  | 1.000 | *0.533 | *0.494 | *0.757 | *0.775 | *0.753 |
|  |  |  |  |  |  |  |  | (0.001) | (0.002) | (<0.001) | (<0.001) | (<0.001) |
| ΔU-G-NS |  |  |  |  |  |  |  | 1.000 | *0.309 | *0.578 | *0.413 | *0.474 |
|  |  |  |  |  |  |  |  |  | (0.067) | (<0.001) | (0.012) | (0.004) |
| ΔU-G(6S)-NAc |  |  |  |  |  |  |  |  | 1.000 | *0.285 | *0.422 | *0.500 |
|  |  |  |  |  |  |  |  |  |  | (0.092) | (0.010) | (0.002) |
| ΔU-G-(6S)-NS |  |  |  |  |  |  |  |  |  | 1.000 | *0.753 | *0.621 |
|  |  |  |  |  |  |  |  |  |  |  | (<0.001) | (<0.001) |
| ΔU(2S)-G-NS |  |  |  |  |  |  |  |  |  |  | 1.000 | *0.693 |
|  |  |  |  |  |  |  |  |  |  |  |  | (<0.001) |
| ΔU(2S)-G(6S)-NS |  |  |  |  |  |  |  |  |  |  |  | 1.000 |

Values in each square are the Spearman correlation coefficient, and below in brackets the Probability > |r| under H0:Rho=0.

The number of observations is n =24 except where *indicates n = 36.

**Supplemental Table 3**

Spearman correlation coefficients between saccharide concentrations using all data from theca and granulosa cells from all follicles examined.

|  | **Chondroitin saccharides** | | | | | **Hyaluronan**  **saccharide** | **Heparan saccharides** | | | | | |
| --- | --- | --- | --- | --- | --- | --- | --- | --- | --- | --- | --- | --- |
| **Saccharides** | ∆DiOS | ∆Di4S | ∆Di6S | ∆Di4,6S | ∆Di2,4,6S | ∆DiHA | ΔU-G-NAc | ΔU-G-NS | ΔU-G(6S)-NAc | ΔU-G-(6S)-NS | ΔU(2S)-G-NS | ΔU(2S)-G(6S)-NS |
| ∆DiOS | 1.000 | 0.616 | 0.781 | 0.002 | 0.324 | 0.172 | 0.142 | 0.080 | 0.400 | 0.087 | 0.233 | 0.149 |
|  |  | (0.033) | (0.003) | (0.995) | (0.304) | (0.593) | (0.660) | (0.804) | (0.198) | (0.788) | (0.466) | (0.644) |
| ∆Di4S |  | 1.000 | 0.615 | 0.445 | 0.389 | 0.500 | 0.585 | 0.389 | 0.477 | 0.558 | 0.548 | 0.574 |
|  |  |  | (0.033) | (0.147) | (0.212) | (0.098) | (0.046) | (0.212) | (0.117) | (0.059) | 0.065 | 0.051 |
| ∆Di6S |  |  | 1.000 | 0.154 | 0.518 | 0.560 | 0.284 | 0.257 | 0.174 | 0.236 | 0.476 | 0.374 |
|  |  |  |  | (0.633) | (0.084) | (0.058) | (0.372) | (0.421) | (0.588) | (0.461) | 0.118 | 0.231 |
| ∆Di4,6S |  |  |  | 1.000 | 0.694 | 0.562 | 0.365 | 0.227 | 0.426 | 0.358 | 0.328 | 0.320 |
|  |  |  |  |  | (0.012) | (0.057) | (0.244) | (0.478) | (0.167) | (0.253) | 0.297 | 0.311 |
| ∆Di2,4,6S |  |  |  |  | 1.000 | 0.622 | 0.195 | -0.033 | 0.340 | 0.168 | 0.269 | 0.202 |
|  |  |  |  |  |  | (0.031) | (0.544) | (0.919) | (0.279) | (0.602) | 0.398 | 0.529 |
| ∆DiHA |  |  |  |  |  | 1.000 | 0.381 | 0.217 | 0.260 | 0.395 | 0.436 | 0.454 |
|  |  |  |  |  |  |  | (0.221) | (0.498) | (0.415) | (0.204) | 0.156 | 0.139 |
| ΔU-G-NAc |  |  |  |  |  |  | 1.000 | *0.591 | *0.484 | *0.702 | *0.649 | *0.653 |
|  |  |  |  |  |  |  |  | (<0.001) | (0.003) | (<0.001) | <0.001 | <.0001 |
| ΔU-G-NS |  |  |  |  |  |  |  | 1.000 | *0.446 | *0.629 | *0.574 | *0.564 |
|  |  |  |  |  |  |  |  |  | (0.006) | (<0.001) | (<0.001) | (<0.001) |
| ΔU-G(6S)-NAc |  |  |  |  |  |  |  |  | 1.000 | *0.346 | *0.320 | *0.376 |
|  |  |  |  |  |  |  |  |  |  | (0.039) | (0.057) | (0.024) |
| ΔU-G-(6S)-NS |  |  |  |  |  |  |  |  |  | 1.000 | *0.600 | *0.660 |
|  |  |  |  |  |  |  |  |  |  |  | (<0.001) | (<0.001) |
| ΔU(2S)-G-NS |  |  |  |  |  |  |  |  |  |  | 1.000 | *0.680 |
|  |  |  |  |  |  |  |  |  |  |  |  | (<0.001) |
| ΔU(2S)-G(6S)-NS |  |  |  |  |  |  |  |  |  |  |  | 1.000 |

Values in each square are the Spearman correlation coefficient, and below in brackets the Probability > |r| under H0:Rho=0.

The number of observations is n =36 except where *indicates n = 12.
